# RSPOs facilitated HSC activation and promoted hepatic fibrogenesis

**DOI:** 10.18632/oncotarget.11654

**Published:** 2016-08-27

**Authors:** Xinguang Yin, Huixing Yi, Linlin Wang, Wanxin Wu, Xiaojun Wu, Linghua Yu

**Affiliations:** ^1^ Centre for Gastroenterology and Hepatology, The Maternity and Child Health Care Hospital affiliated to Jiaxing College, Jiaxing, 314001, Zhejiang Province, PR China; ^2^ Centre for Gastroenterology and Hepatology, The First Affiliated Hospital of Jiaxing College, Jiaxing, 314001, Zhejiang Province, PR China; ^3^ Intensive Care Unit, The Second Affiliated Hospital of Zhejiang University, Hangzhou, 310009, Zhejiang Province, PR China; ^4^ Department of Basic Medicine Sciences, School of Medicine, Zhejiang University, Hangzhou, 310058, China; ^5^ Deparment of Pathology, The First Affiliated Hospital of Jiaxing College, Jiaxing, 314001, Zhejiang Province, PR China

**Keywords:** RSPO, hepatic fibrosis, Wnt pathway, HSC

## Abstract

Roof plate-specific spondin (RSPO) proteins are potent Wnt pathway agonists and involve in a broad range of developmental and physiological processes. This study investigated the activities and mechanisms of RSPOs in liver fibrogenesis, especially in hepatic stellate cell (HSC) activation. HSC activation was assessed by fibrosis biomarker (α-smooth muscle actin and Collagen-I), phenotypic change (accumulation of lipid droplets), and increased proliferation. Similarly, Wnt pathway activity was evaluated by the expression of nuclear β-catenin and T cell-specific transcription factors (TCF) activity. We found RSPOs were overexpressed in human fibrotic liver tissue and the expressions were correlated with liver fibrosis stages. *In vitro* studies showed RSPOs level increased during HSC activation, and stimuli with RSPOs enhanced Wnt pathway activity and promoted HSC activation subsequently. Furthermore, *in vivo* experiments demonstrated that the knockdown of RSPOs suppressed both Wnt pathway activity and HSC activation. Interestingly, the inhibitor of the Wnt signaling pathway Dickkopf1 impairs RSPOs effects on HSCs. Taken together, our results revealed that RSPOs facilitated HSC activation and promote liver fibrogenesis by enhancing the Wnt pathway.

## INTRODUCTION

Hepatic fibrosis is a reversible wound-healing response to chronic liver injury, and its advanced stage, cirrhosis, is among the most common cause of death worldwide [1–3]. Even worse, patients with cirrhosis are at the highest risk of developing hepatocellular carcinoma (HCC), the sixth most prevalent cancer and the major cause of cancer-related death [4]. Currently, the optimal management of liver fibrosis depends on early diagnosis. However, the early stages of hepatic fibrosis are asymptomatic, hence make liver fibrosis a tremendous medical challenge.

Hepatic stellate cell (HSC) activation and perpetuation is the central event of liver fibrogenesis [1, 2]. Intracellular events driving HSC activation and perpetuation are increasing being reported, among which the Wnt pathway has been revealed as playing a pivotal role [5–7]. Wnt signaling are crucial for tissue renewal and repair during homeostasis [8, 9]. It is proven that Roof plate-specific spondin (RSPOs) strongly potentiate the Wnt pathway, especially by enhancing the transcriptional activity of β-catenin [10, 11]. RSPO protein family consists of four homologous members (RSPO1, RSPO2, RSPO3, and RSPO4) that are evolutionarily conserved in vertebrate and involved in a broad range of developmental and physiological processes [10, 11]. Prior studies have documented that RSPO1-LGR5 (Leucine-rich repeat-containing G-protein coupled receptor 5) axis is crucial to the liver stem cell upon tissue damage [12], and RSPO1 is critical for intestinal stem cell induction [13, 14]. Moreover, RSPO2 and RSPO3 are correlated with colon cancer [15, 16]. Above studies indicate RSPOs are essential in regulating cell proliferation, differentiation, and survival during embryonic development and tissue homeostasis. However, the connection between RSPOs and liver fibrosis is not extensively studied.

In this study, we observed high expressions of RSPOs (RSPO1, RSPO2, and RSPO3) in human liver fibrotic tissue and the expressions were correlated with hepatic fibrosis stages. *In vitro* studies showed RSPOs level increased during HSC activation and co-cultured with RSPOs promoted HSC activation. Furthermore, knockdown of RSPOs in mice via lentivirus-delivery suppressed both Wnt pathway activity and HSC activation. Additionally, Dickkopf1 (DKK1), a well-known Wnt inhibitor, suppressed RSPOs' effect on HSC activation. Our study showed that RSPOs promote liver fibrogenesis and indicated RSPOs maybe a potential target for early-diagnosis and therapeutic intervention of hepatic fibrosis.

## RESULTS

### Overexpression of RSPOs in human fibrotic liver tissues

**Figure 1:**
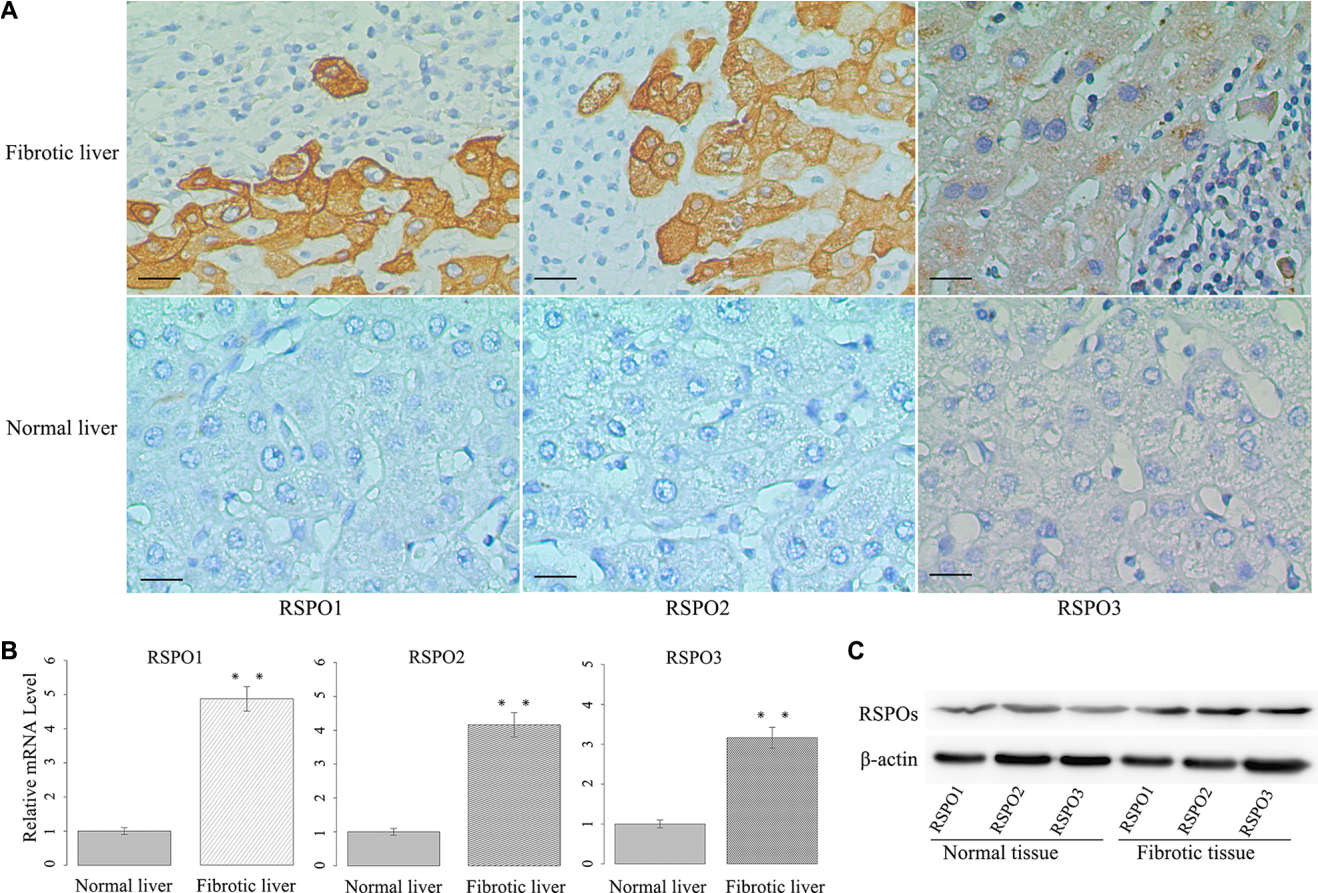
RSPOs (RSPO1, RSPO2, and RSPO3) were overexpressed in human fibrotic liver tissues (**A**) Representative pictures of immunostaining showed RSPOs expression were positive in human fibrotic liver tissue, whereas they were negative in human normal liver tissue (bar = 50 μm, magnification × 400). (**B**) The mRNA levels of RSPOs were significantly higher in human fibrotic liver tissue (*n* = 32) compared with human normal liver tissue (*n* = 10). (**C**) Western Blot assay indicated RSPOs were overexpressed in human fibrotic liver tissue. Data represents the mean of three independent experiments, and error bars are standard deviation of means. **p < 0.01 compared with the human normal liver tissue.

We evaluated RSPOs protein expression in clinical samples of hepatic fibrosis. A total of sixty-three human fibrotic liver tissue samples were examined using immunohistochemical staining with antibodies against human RSPO1, RSPO2, and RSPO3, respectively. Ten normal liver tissue samples were served as the control. Samples were considered positive if either cell nucleus or cytoplasm stained positive. As shown in Figure 1A (representative pictures), the staining of RSPOs were mostly found in the cytoplasm of HSCs. A strong expression of RSPO1 in fibrotic liver tissues (score 2 or 3) was found in 36 of 63 patients (57.1%), whereas a weak immunoreactivity (score 0 or 1) was detected in other patients (42.9%). However, the expression of RSPO1 in normal liver tissues was significantly lower than that in fibrotic tissues (Wilcoxon Signed Ranks Test, *p* = 0.019). Similar expression patterns were found in the staining of RSPO2 and RSPO3 too. The strong and weak expressions of RSPO2 in fibrotic tissues were 38 (60.3%) and 25 (39.7%) respectively, whereas a significantly lower expression of RSPO2 was found in normal tissues (Wilcoxon Signed Ranks Test, *p* = 0.012). For RSPO3, the strong and weak expressions in fibrotic tissues were 38 (60.3%) and 25 (39.7%) respectively, while the expression of RSPO3 in normal tissues was significantly lower than that in fibrotic tissues (Wilcoxon Signed Ranks Test, *p* = 0.004).

To verify this finding, the mRNA levels of RSPOs in human fibrotic liver tissue were examined by real-time PCR. Thirty-two human fibrotic liver tissue samples were examined, with ten normal liver tissue samples serving as the control. The mRNA levels of RSPOs were significantly higher in fibrotic tissues (*p* < 0.01 for RSPO1, RSPO2, and RSPO3) than those in normal tissues (Figure 1B). The protein expressions of RSPOs in the liver tissue were analyzed by Western blot assay. The results of the Western blot assay (Figure 1C) were consistent with those of the PCR experiments, that the protein expressions of RSPOs increased in the fibrotic tissue.

### The expressions of RSPOs were correlated with liver fibrosis stages

Correlations between RSPOs expression and the clinicopathological characteristics of liver fibrosis were analyzed. As shown in Table 1, no correlation was found between the expression of RSPOs and patient age (Mann-Whitney *U* Test, *p* = 0.826 for RSPO1, *p* = 0.955 for RSPO2, and *p* = 0.113 for RSPO3) or gender (Mann-Whitney *U* Test, *p* = 0.638 for RSPO1, *p* = 0.705 for RSPO2, and *p* = 0.536 for RSPO3). Interestingly, expressions of RSPOs were strongly correlated with the modified Knodell score for liver fibrosis stages (Kruskal-Wallis Rank Sum Test, *p* < 0.01 for RSPO1, RSPO2, and RSPO3) [17].

**Table 1:** Clinicopathological characteristics and RSPOs expression of liver fibrosis patients

Characteristics	Patients number	RSPO1			RSPO2			RSPO3		
		Weak	Strong	*P* value	Weak	Strong	*P* value	Weak	Strong	*P* value
Age										
≤ 50	18	8	10	0.826	7	11	0.955	11	7	0.113
> 50	45	19	26		18	27		14	31	
Gender										
Male	37	18	19	0.638	17	20	0.705	15	22	0.536
Female	26	9	17		8	18		10	16	
Knodell score										
I	11	10	1		8	3		8	3	
II	17	12	5	< 0.01	10	7	< 0.01	13	4	< 0.01
III	23	5	18		7	16		4	19	
IV	12	0	12		0	12		0	12	

### RSPOs protein and mRNA increased during the culture-initiated activation of HSCs

**Figure 2:**
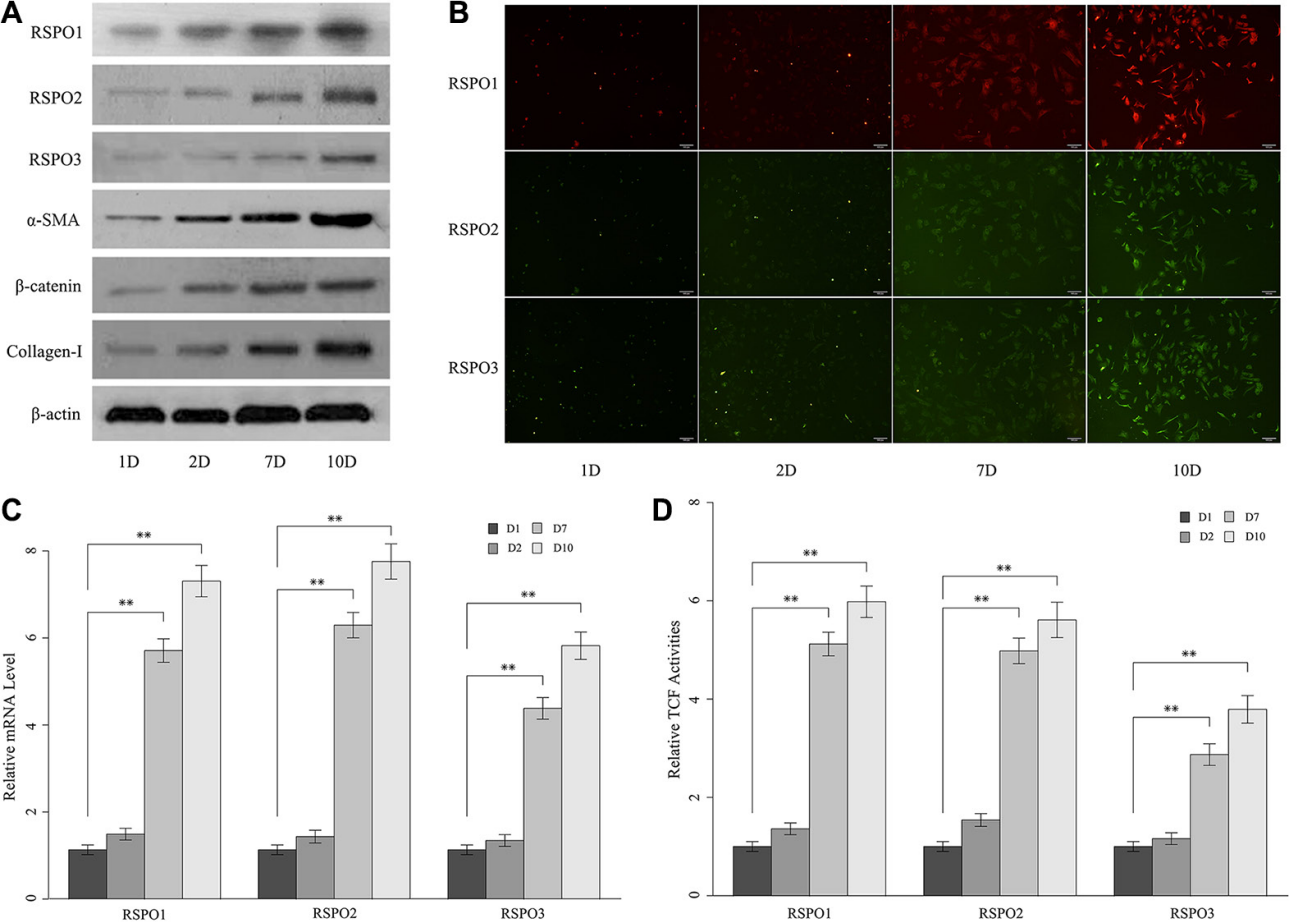
The expressions of RSPOs (RSPO1, RSPO2, and RSPO3) were increased during the culture-initiated activation of mice HSCs (**A**) Western blot assay showed the protein expression of RSPOs, α-SMA, nuclear β-catenin, and Collagen-I increased over time. (**B**) Immunofluorescence results were consistent with the findings of WB experiments. (**C**) The mRNA levels of RSPOs were significantly higher in the fully activated HSCs (day 7 and day 10) compared with the quiescent HSCs (day 1); and there's no significant difference in mRNA level between day 1 and day 2. (**D**) TCF activities elevated in the fully activated HSCs (day 7 and day 10), and there's no obvious change between day 1 and day 2. Data represents the mean of three independent experiments, and error bars are standard deviation of means. *p < 0.05 compared with the quiescent HSCs (day 1), **p < 0.01 compared with the quiescent HSCs.

We further analyze the expressions of RSPOs during the culture-initiated activation of mice HSCs. Freshly isolated mice HSCs were cultured *in vitro* for one day, two days, seven days, and 10 days. Then the protein expressions of RSPOs (RSPO1, RSPO2, and RSPO3), fibrosis biomarker α-smooth muscle actin (α-SMA) and Collagen-I (Figure 2A) were examined by Western blot assay. Results showed that the protein expression of α-SMA and Collagen-I were notably increased in day 7 compared with day 1 and day 2, which indicated that the freshly isolated HSCs were undergoing a phenotypic switch from quiescent to highly fibrogenic cells. Intriguingly, protein expressions of α-SMA and Collagen-I were kept high in day 10, which implied perpetuation of the activated HSCs. Similarly, protein expressions of RSPOs were remarkably elevated in the fully activated HSCs (day 7 and day 10). The similar results were also observed by immunofluorescence analysis (Figure 2B). To confirm the above findings, the mRNA levels of RSPOs (Figure 2C) were tested by real-time PCR. We found that the mRNA levels of RSPOs have no obvious changes between day 1 and day 2, whereas there's significant increase between day 7 and day 1 (*p* < 0.01 for RSPO1, RSPO2, and RSPO3) and day 10 and day 1 (*p* < 0.01 for RSPO1, RSPO2, and RSPO3).

WNT/β-catenin pathway activities during HSCs culture-initiation were analyzed. The expression of nuclear β-catenin was prominently up-regulated in the fully activated HSCs (day 7 and day 10) compared with the quiescent HSCs (day 1 and day 2) (Figure 2A). Similarly, TCF activities significantly elevated on day 7 and day 10, while there's no notably change between day 1 and day 2 (Figure 2D). These findings suggested stabilization and translocation of cytoplasmic β-catenin into the nucleus and subsequent activation of TCF/LEF (Lymphoid enhancer-binding factors)-dependent transcription of the fully activated HSCs.

### Exogenous stimulation with recombinant RSPOs enhanced WNT pathway activities and subsequently promoted HSCs activation

**Figure 3:**
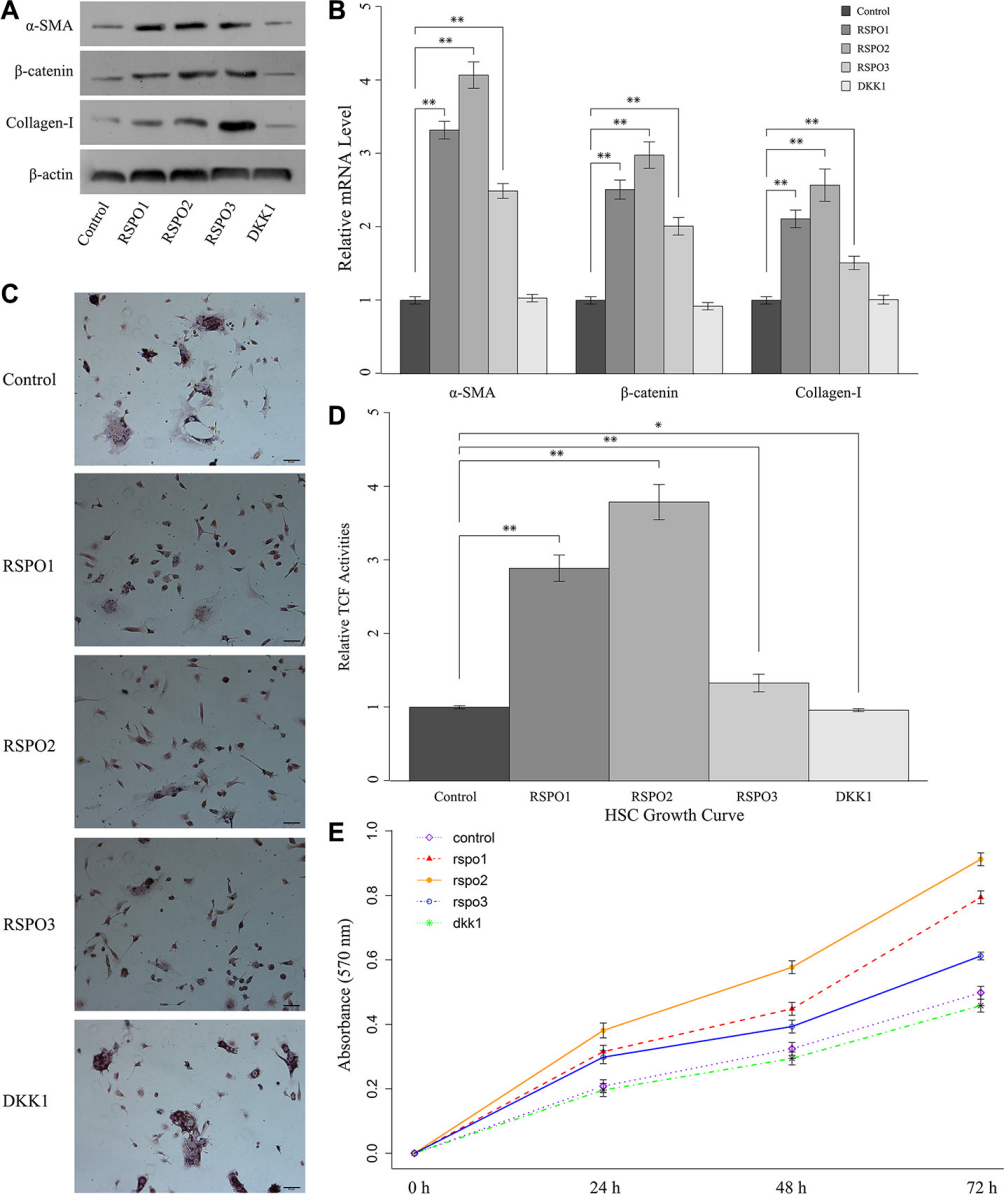
RSPOs (RSPO1, RSPO2, and RSPO3) enhanced WNT pathway activity and subsequently promoted HSCs fibrogenesis and proliferation, whereas DKK1 repressed HSCs activation (**A**) The protein expression of α-SMA, nuclear β-catenin, and Collagen-I in freshly isolated HSCs up-regulated with the stimulation of recombinant RSPOs, whereas recombinant DKK1 led to the down-regulation of α-SMA, nuclear β-catenin, and Collagen-I in activated HSCs. (**B**) Real-time PCR results were consistent with the findings of the Western blot assay. (**C**) Oil red O staining demonstrated the lipid droplets were reduced in HSCs when treated with recombinant RSPOs, whereas there's no obvious change with the stimulation of recombinant DKK1 (bar = 50 μm, magnification × 400). (**D**) TCF activities significantly increased when treated with recombinant RSPOs compared with the control, and decreased when treated with recombinant DKK1. (**E**) MTT assay showed RSPOs up-regulate HSCs proliferation, whereas DKK1 suppresses HSCs proliferation. Data represents the mean of three independent experiments, and error bars are standard deviation of means. *p < 0.05 compared with the control (quiescent HSCs with no treatment of recombinant RSPOs and DKK1), **p < 0.01 compared with the control.

To define the function of RSPOs in liver fibrogenesis, freshly isolated mice HSCs were co-cultured with recombinant RSPOs (RSPO1, RSPO2, and RSPO3). Quiescent HSCs with no treatment served as the control. The protein expressions (Figure 3A) and mRNA levels (Figure 3B) of α-SMA, nuclear β-catenin, and Collagen-I were examined by Western blot assay and real-time PCR, respectively. The mRNA level of α-SMA, nuclear β-catenin, and Collagen-I were significantly increased when co-cultured with RSPOs (*P* < 0.01 for RSPO1, RSPO2, and RSPO3). Accordingly, the protein expression of α-SMA, nuclear β-catenin, and Collagen-I were notably enhanced when stimulated by recombinant RSPOs.

To understand the role of RSPOs in the activation of WNT/β-catenin pathway and subsequent HSCs activation, TCF activities under the stimulation of RSPOs (Figure 3D) were analyzed by Luciferase reporter assay. Experiment results were consistent with the above findings that TCF activities significantly increased when treated with recombinant RSPOs (*p* < 0.01 for RSPO1, RSPO2, and RSPO3).

Following liver injury, HSCs undergo a phenotypic transition from a quiescent, vitamin A-storing cell into a myofibroblast-like cell lacking cytoplasmic lipid droplets [1]. Oil red O staining demonstrated that the lipid droplets were reduced in HSCs co-cultured with recombinant RSPOs (Figure 3C), which was in accordance with the above findings that RSPOs promote the HSCs activation.

Previous studies showed the activation of HSC induces a proliferative response [1]. Results of proliferation assay showed that HSCs growth significantly increased when treated with RSPOs (Figure 3E). Collectively, these findings suggested that co-culture with RSPOs enhanced WNT pathway activities and subsequently facilitated HSCs activation.

### DKK1 impacted HSCs activation

In order to understand if Wnt/beta-catenin pathway is the mediator of the activatory and pro-fibrogenic effects of RSPOs we treated activated mice HSCs with the recombinant DKK1, and quiescent HSCs with no treatment served as the control. The protein expressions (Figure 3A) and mRNA levels (Figure 3B) of α-SMA, nuclear β-catenin, and Collagen-I were examined by Western blot assay and real-time PCR, respectively. Results showed there's no obvious change in both the mRNA level and protein expression of α-SMA, nuclear β-catenin, and Collagen-I compared with the control. Oil red O staining (Figure 3C) presented increased lipid droplets in HSCs cytoplasm. Luciferase reporter assay demonstrated there's significant decrease in the TCF activity (*p* = 0.012) when treated with DKK1 (Figure 3D). And the HSCs growth curve was almost same as the control (Figure 3E). These findings indicated DKK1 might inhibit RSPOs' effects on HSCs activation by suppressing the Wnt signaling activity.

### The knockdown of RSPOs repressed WNT pathway activity and HSCs activation

**Figure 4:**
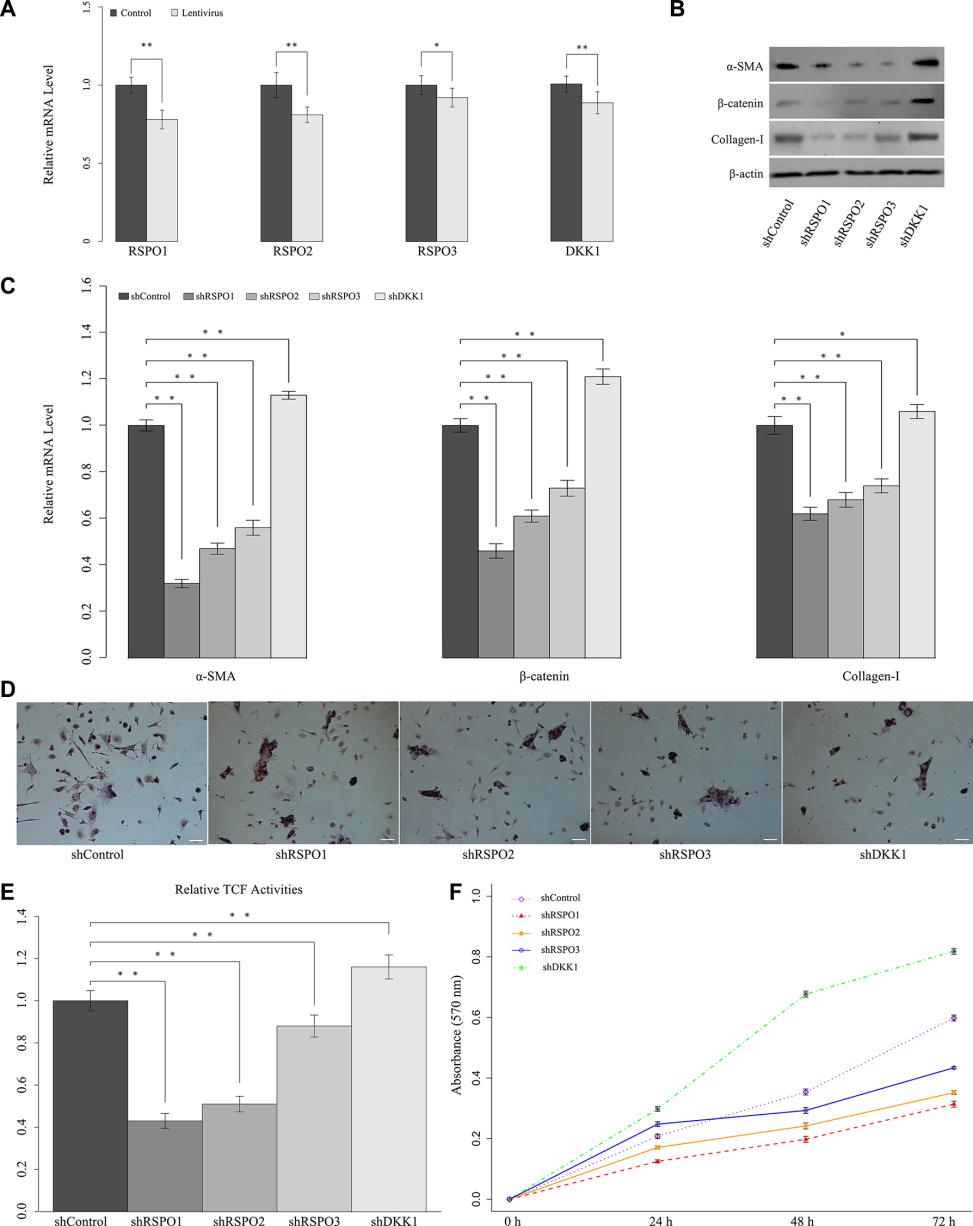
Knockdown of RSPOs (RSPO1, RSPO2, and RSPO3) repressed WNT pathway activity and HSCs activation, whereas knockdown of DKK1 enhanced RSPOs' expression (**A**) Real-time PCR results showed that lentivirus delivery decreased the mRNA level of both RSPOs and DKK1. (**B**) Knockdown of RSPOs down-regulated the expression of α-SMA, nuclear β-catenin, and Collagen-I compared with the control, whereas knockdown of DKK1 enhanced the expression of α-SMA, nuclear β-catenin, and Collagen-I. (**C**) Real-time PCR results were consistent with the findings of Western blot assay. (**D**) Oil Red O staining showed that knockdown of RSPOs increased the lipid droplets in HSCs, while knockdown of DKK1 reduced the lipid droplets (bar = 50 μm, magnification × 400). (**E**) TCF activities significantly decreased in RSPOs-knockdown HSCs compared with the control, while DKK1-knockdown significantly increased TCF activities. (**F**) MTT assay showed RSPOs-knockdown suppressed HSCs proliferation, whereas DKK1-knockdown promoted HSCs proliferation. Data represents the mean of three independent experiments, and error bars are standard deviation of means. *p < 0.05 compared with the control (CCl4-induced hepatic fibrosis mice transfected with scrambled siRNA through Lentivirus transduction), **p < 0.01 compared with the control.

To get a comprehensive understanding of the biological role of RSPOs in liver fibrogenesis and the underlying mechanism of the above findings, RSPOs gene silence was achieved by lentivirus delivery. The effect of lentivirus delivery on RSPOs expression were verified by real-time PCR. Results showed that the mRNA level of both RSPOs and DKK1 are decreased (Figure 4A). To further elucidate the mechanism responsible for RSPOs-induced HSCs activation, *in vivo* lentivirus-delivery on mice was carried out. CCl_4_-induced hepatic fibrosis mice were divided into five groups (*n* = 12 for each group). Each group was transfected with one of the lentiviral vectors (Lenti-shRSPO1, Lenti-shRSPO2, Lenti-shRSPO3, Lenti-shDKK1, and Lenti-shControl serving as the control) through tail vein injections. Mice HSCs were harvested for further analysis. The protein expressions (Figure 4B) and mRNA levels (Figure 4C) of α-SMA, nuclear β-catenin, and Collagen-I were examined by Western blot assay and real-time PCR, respectively. The mRNA levels of α-SMA, nuclear β-catenin, and Collagen-I were significantly lower in shRSPOs groups (*p* < 0.01 for shRSPO1, shRSPO2, and shRSPO3) than those in the control group. In contrast, the mRNA levels of α-SMA (*p* < 0.01), nuclear β-catenin (*p* < 0.01), and Collagen-I (*p* = 0.022) were significantly higher in shDKK1 groups than those in the control group. Results of Western blot assay were in accordance with those of PCR tests, that the protein expressions of α-SMA, nuclear β-catenin, and Collagen-I decreased in shRSPOs groups, whereas they increased in shDKK1 group.

Oil red O staining presented lipid droplets increased in shRSPOs (shRSPO1, shRSPO2, and shRSPO3), which suggested the activated HSCs might be in the process of reversion to a more quiescent phenotype (Figure 4D). Luciferase reporter assay showed there's a significant decrease in the TCF activity in shRSPOs groups (*p* < 0.01 for shRSPO1, shRSPO2, and shRSPO3) (Figure 4E). And the HSCs growth rate of shRSPOs groups notably dropped compared with the control (Figure 4F). Overall, these findings suggested the knockdown of RSPOs gene suppresses WNT pathway activities and subsequently lead to liver fibrosis resolution.

Interestingly, our study showed DKK1 impairs RSPOs effects on HSCs. In consistent with the results of PCR and Western blot assay, Oil red O staining showed lipid droplets decreased in shDKK1 group (Figure 4D), and there's significant increase in the TCF activity in shDKK1 groups (*p* < 0.01) (Figure 4E). Moreover, the HSCs growth rate of shDKK1 groups dramatically up-regulated compared with the control (Figure 4F). Collectively, above findings indicated the synergistic effects of RSPOs and DKK1 might facilitate the HSC activation.

## DISCUSSION

The development of hepatic fibrosis is the essential stage towards mortal complications of liver diseases, such as cirrhosis and HCC. While liver fibrosis, and even cirrhosis in early stages, can be reversed by controlling the underlying cause, the only treatment currently available for patients with final-stage cirrhosis is transplantation [18]. Meanwhile, prior studies reported that the bidirectional interactions between tumors and HSCs compose an “amplification loop” to further promote metastatic growth in the liver [19]. It thus makes early diagnosis and therapeutic intervention of liver fibrosis a major unmet medical need.

In this study, our data provided evidence that RSPOs may contribute to the hepatic fibrogenesis. Analysis with human liver fibrotic tissue showed that RSPOs are overexpressed comparing with the normal liver tissues, and the expressions of RSPOs are correlated with liver fibrosis stages. These findings make RSPOs a compelling target for early-detection and treatment of hepatic fibrosis.

*In vivo* study showed the RSPOs expressions increase during HSC activation and the fully activated HSCs keep RSPOs level high, which indicated initiation following by perpetuation in the activated HSCs. These findings were in accordance with a prior study that the transcriptional level of RSPOs increased three to six fold following liver insults [12].

Co-cultured with recombinant RSPOs enhanced Wnt pathway activities and promoted HSCs fibrogenesis, proliferation, and the phenotype switch, while knockdown of RSPOs repressed Wnt pathway activities and inhibited HSCs activation. Furthermore, our study also indicated DKK1 has an inhibitory effect on RSPOs functions. It has been reported that DKK1 inhibits RSPOs functions by competing for LRP5 (low-density lipoprotein receptor-related protein 5) or LRP6 receptors [20–22], and the balance between RSPOs and DKK1 orchestrates tissue homeostasis [23]. Our data was consistent with those reports and suggested that the interplay of RSPOs and DKK1 coordinates hepatic fibrogenesis *in vivo* and *in vitro*.

**Figure 5:**
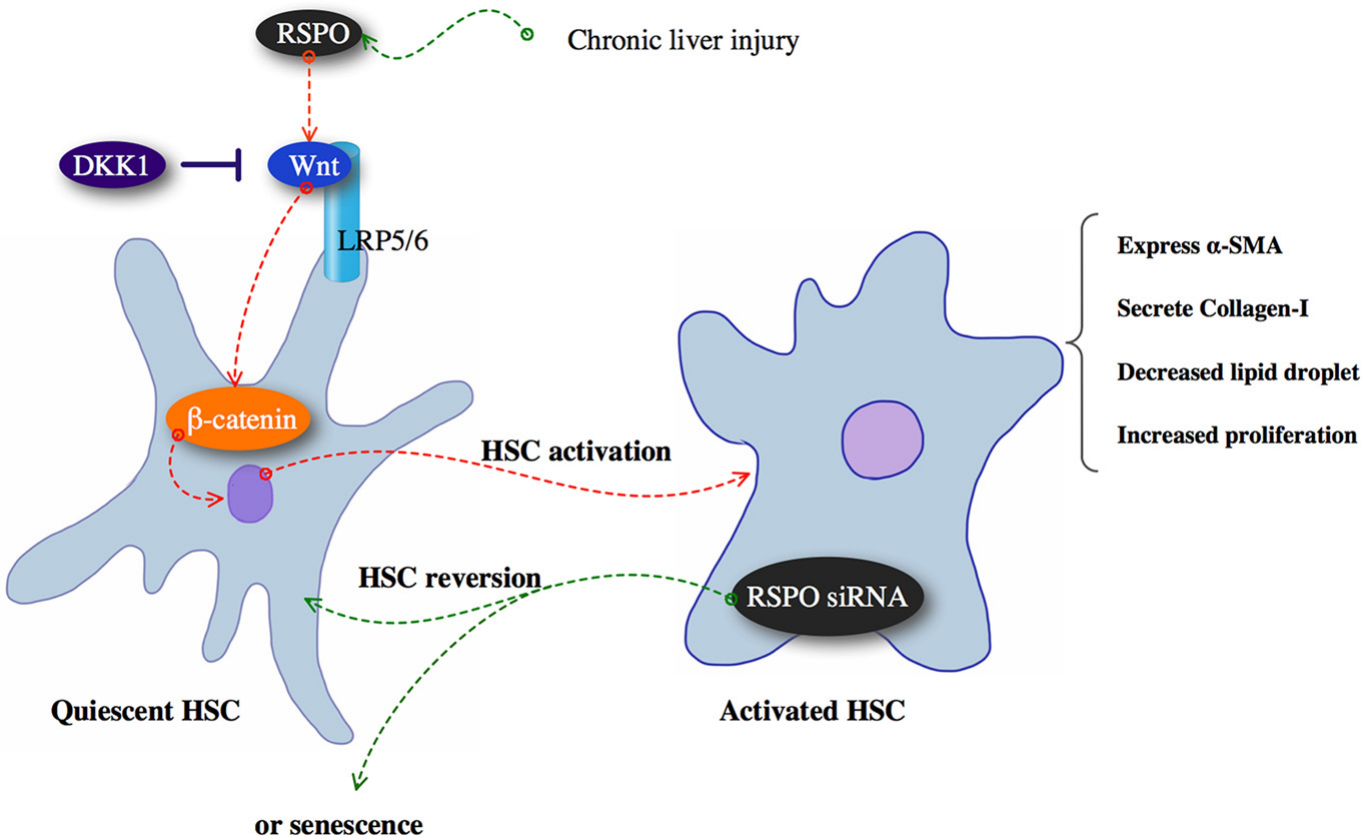
Chronic liver injury might up-regulate RSPOs expression and subsequently promoted hepatic fibrogenesis by enhancing Wnt/β-catenin pathway, whereas DKK1 has the inhibitory effect on RSPOs function

Taken together, our study revealed RSPOs promote hepatic fibrogenesis by enhancing Wnt pathway, whereas DKK1 has the inhibitory effect on RSPOs function (Figure 5). Since hepatic fibrosis is the fundamental stage towards mortal liver diseases, limiting the progression of fibrogenesis without impacting the wound healing still remains a challenge for physicians and researchers. Our study might bring new angles to the molecular mechanism responsible for the liver fibrosis.

Previous researches report detecting DKK1 in serum as a biomarker for the diagnosis of HCC [24]. Similarly, to apply our findings to search for better markers of fibrosis, further studies are required to investigate the feasibility of trace detection of RSPOs proteins in serum and connecting them with the stages of liver fibrosis.

## MATERIALS AND METHODS

### Patients and liver tissue samples

The study was approved by the Ethics Committee of Jiaxing College. Human liver tissues were obtained from the First Affiliated Hospital of Jiaxing College, with written informed consent from each patient. A total of 63 fibrotic liver tissue samples were collected, with ten normal hepatic tissue samples served as the control. Tissue samples were preserved at the temperature of −80°C.

### Liver fibrosis evaluations

A modified Knodell scoring system was used to evaluate the fibrotic stages in the human liver samples [17]. Two experienced pathologists evaluated the liver histology of the samples in a double-blinded manner. Results were graded in four categories: fibrous portal expansion (I), fibrous septa (II), bridging fibrosis (III), and cirrhosis (IV).

### Animals used

Healthy Kunming mice (8–10 weeks old, body weight, 38–40 g) were purchased from Shanghai SLAC Laboratory Animal Co Ltd (Shanghai, China). The animals were housed under standard animal laboratory conditions and supplied with laboratory chow and water. All individuals involved in animal research received instructions in experimental methods and in the care, maintenance, and handling of mice. All institutional and national guidelines for the care and use of laboratory animals were followed. The protocol of the experiments was approved by the Committee on the Ethics of Animal Experiments of Jiaxing College.

### Antibodies and reagents

Rabbits polyclonal to RSPO1/2/3, α-SMA, and Collagen-I were purchased from Abcam (Cambridge, MA, USA). Rabbits polyclonal to β-catenin, HRP-conjugated secondary anti-rabbit IgG were obtained from CST (Cell Signaling Technology, MA, USA). Human RSPO1/2/3 antibody, recombinant mouse RSPOs (RSPO1, RSPO2, and RSPO3), and recombinant mouse DKK1 were purchased from R&D (R&D Systems, Minneapolis, MN, USA). NEPER Nuclear and Cytoplasmic Extraction Reagent Kit was obtained from Pierce (Pierce Biotechnology, Rockford, IL, USA). Lipofectamine 2000, Opti-MEM I Reduced Serum Medium, and TRIzol were obtained from Invitrogen (Life Technologies, Carlsbad, CA, USA). DMEM and FBS were purchased from Gibco (Life Technologies, Carlsbad, CA, USA). TCF reporter plasmid TOPFLASH and the control inactive reporter FOPFLASH was purchased from Upstate (Upstate Biotechnology Inc., Lake Placid, NY, USA). CCl4, olive oil, collagenase IV, pronase E, Nycodenz, DAPI, BSA, Triton X-100, MTT, and DMSO were obtained from Sigma-Aldrich (Sigma-Aldrich, St. Louis, MO, USA). Lentiviral vector was purchased from Inovogen (Inovogen Tech, Beijing, China). Oil Red O kit was purchased from AMTS (American Mastertech Scientific; Lodi, CA, USA).

### HSC isolation and culture

Primary stellate cells were isolated from the livers of Kunming mice as previously described [25]. Briefly, mouse liver was perfused *in situ* with DMEM (Gibco, United States) to purge the liver of blood. After digestion of the liver with pronase E (Sigma, United States) and collagenase IV (Sigma, United States), dispersed cell suspensions were layered by Nycodenz (Sigma, United States) density gradient centrifugation according to manufactory's protocol. The resulting upper layer contained the freshly isolated HSCs. The purity of isolated HSCs was examined by phase-contrast microscopy, and viability based on trypan blue exclusion (purity > 95%, viability > 95%). The isolated HSCs were then cultured on uncoated plastic plates in DMEM (Gibco, United States) supplemented with 10% FBS (Gibco, United States) at 37°C at a density of 1 × 10^5^ cells/cm^2^ for future research.

### Immunohistochemical staining

Tissue samples were fixed in 10% formalin and paraffin-embedded for immunohistochemical analysis. After deparaffinization, rehydration, and antigen retrieval, liver tissue sections were incubated with 3% H_2_O_2_, and followed by serum blocking with 10% goat serum in 5% bovine serum albumin (Sigma, United States). Then, liver tissue sections were incubated with the primary antibody against RSPO1 (1:1000 dilution, R&D Systems, United States), RSPO2 (1:1000 dilution, R&D Systems, United States), and RSPO3 (1:1000 dilution, R&D Systems, United States) at 4°C overnight, followed by HRP-conjugated secondary anti-rabbit IgG (1:2000 dilution, Santa Cruz, United States) antibody for 1 h at room temperature. Immunohistochemical staining was carried out using SABC kit (Boster, China). For quantification of the immunostain, cells were counted in six randomly chosen high-power fields at 400-fold magnification by two experienced researchers in a double-blinded manner. Results were scored by the percentage of RSPOs-stained cells as below: < 10% (score 0), 10%~50% (score 1), 51%~75% (score 2), and 76%~100% (score 3). Score 0 and 1 were considered as the weak expression, whereas score 2 and 3 were the positive expression.

### Immunofluorescence analysis

Cells were paraformaldehyde-fixed followed by permeabilization with 0.1% Triton X100 in PBS and blocked with 4% BSA. Then cells were incubated with rabbit polyclonal antibody for RSPOs (Abcam, Cambridge, MA) overnight at 4°C. Cells were then incubated with HRP-conjugated rabbit IgG (CST, MA, USA) for one hour. Nuclei was stained with DAPI (Sigma-Aldrich, MO, USA). Images were captured using an epifluorescence microscope (Olympus, Tokyo, Japan).

### Real-time PCR

The mRNA level was quantified by real-time PCR (StepOne-Plus, Applied Biosystems, CA) per the manufacturer's instructions. To minimize the problems associated with DNA contamination, primers were designed to span at least one intron in the genomic sequence. Primers used for amplification were listed in Table 2. Total RNA from the cells was isolated using Trizol (Invitrogen, Carlsbad, CA) according to the manufacturer's protocol. The quality of isolated RNA was assessed at absorbance ratios of A260/A280 (1.8–2.2) and A260/A230 (3.0–4.0) with an UV-1800 spectrophotometer (Shimadzu, Kyoto, Japan). Thermocycling conditions consisted of an initial step of 2 min at 50°C, denaturation of 10 min at 95°C, followed by 35–40 cycles at 95°C for 30 s, 60°C for 45 s, and 72°C for 1 min and a final elongation step at 72°C for 10 min. Each sample was analyzed in triplicate, with β-actin used for normalization. Comparative threshold (Ct) method was used for calculating the relative amount of mRNA of the sample compared with the control.

**Table 2:** Primers sequences

Gene	Forward sequence	Reverse sequence
**R-Spondin1**	5′-ATTCTGCTGGAGAGGAACGA-3′	5′-CTCCTGACACTTGGTGCAGA-3′
**R-Spondin2**	5′-CAAGCATGGACTCAGCGTTA-3′	5′-TCTACAGCCTTGTGCCTCCT-3′
**R-Spondin3**	5′-GTGAGGCCAGTGAATGGAGT-3′	5′-CTCGCTCTCCCTTTGAACAC-3′
**α-SMA**	5′-CTGACAGAGGCACCACTGAA-3′	5′-CATCTCCAGAGTCCAGCACA-3′
**Collagen-I**	5′-TTCACCTACAGCACGCTTGTG-3′	5′-GATGACTGTCTTGCCCCAAGTT-3′
**β-catenin**	5′-CAGCAGTTTGTGGAGGGCGTG-3′	5′-TGTCAGGGGAGCTGTGGCTCC-3′
**β-actin**	5′-TCATCACTATTGGCAACGAGC-3′	5′-AACAGTCCGCCTAGAAGCAC-3′

### Western blot

Nuclear and cytoplasmic protein fractions were extracted using the NE-PER Extraction Reagent Kit (Pierce, United States) according to the manufacturer's instruction. The extracts were then subjected to SDS-PAGE for protein separation and then electrophoretically transferred to nitrocellulose membrane (Axygen, Union City, CA). After blocked by phosphate-buffered saline containing 5% fat-free milk, the nitrocellulose membranes were incubated with rabbit polyclonal antibody for RSPOs (Abcam, Cambridge, MA), α-SMA (Abcam, Cambridge, MA), Collagen-I (Abcam, Cambridge, MA), β-catenin (CST, MA, USA), and β-Actin (HarO, Shanghai, China) overnight at 4°C and then incubated with HRP-conjugated rabbit IgG (CST, MA, USA) for 1.5 h at room temperature. The immuno labeled proteins were detected using a commercial ECL detection kit (HarO, Shanghai, China).

### Stimulation with RSPOs and DKK1

Freshly isolated mice HSCs were maintained until 70% confluence. Then, mediums were changed and HSCs were cultured for 24 h in DMEM (Gibco, United States) with 5% FBS (Gibco, United States). HSCs were then exposed to recombinant mouse RSPOs (100 ng/ml for RSPO1, 10 ng/ml for RSPO2, and 50 ng/ml for RSPO3) (R&D Systems, United States) for 24 h. Similarly, activated HSCs were exposed to recombinant mouse DKK1 (50 ng/ml) (R&D, United States) for 24 h. Finally, cells were harvested for further analysis.

### Luciferase reporter assay

Cells were transiently transfected with TOPFLASH and FOPFLASH (Upstate Biotechnology, United States) using Lipofectamine 2000 (Invitrogen, United States). 24 h after transfection, the cells were harvested and luciferase and renilla luminescence were measured using the Dual-Luciferase Reporter Assay System (Promega, Wisconsin, USA) on a luminometer (BioTek Instruments, USA). TCF reporter activity was presented as by the ratio of firefly to Renilla luciferase activity. Two independent transfections were performed, with each sample tested in triplicate.

### Oil Red O staining

Accumulations of lipid droplets were determined using an Oil red O assay. Briefly, 0.5 g of Oil red O powder (American Mastertech Scientific; CA, USA) was dissolved in 100 mL of isopropanol to make a stock solution, which was diluted with water (3:2) and filtered. Staining was performed at 25°C for 1 h, and plates were then washed twice in ddH_2_O and observed and photographed under a microscope (CK Microscope, Olympus, Tokyo, Japan).

### Growth analysis

HSCs were seeded into 96-well plate at the density of 2.0 × 10^3^ per well. Cell growth was measured by MTT (3-(4,5-Dimethylthiazol-2-yl)-2,5-diphenyltetrazolium bromide) assay at the day 1, day 2, and day 3 after cell seeding. Briefly, 100 μg of MTT (Sigma, United States) was added into each well. After incubation for 4 h at 37°C, purple formazan crystals generated from viable cells were dissolved by adding 100 μl DMSO (Sigma-Aldrich, USA) in each well. The absorbance of each well was then read at 570 nm wavelength in Bio-Rad 550 microplate reader (Bio-Rad, United States). HSCs growth rates were then calculated as below:

Cell growth rates = (average absorbance of test group/control group) × 100%.

### Animal model of hepatic fibrosis

The hepatic fibrosis model of mice was induced by subcutaneous injection of 20% solution of CCL4 in olive oil at a dose of 5 ml/kg twice per week for 10 weeks. Sixty-four Kunming mice were randomly divided into two groups. The first group (*n* = 4) served as a normal control group, and the experimental group (*n* = 60) was hepatic fibrosis models.

### Lentiviral vectors construction and production

shRNA sequences (Table 3) against mouse RSPOs (shRSPO1, shRSPO2, and shRSPO3) and DKK1 (shDKK1) were subcloned into EcoR I and BamH I sites of lentiviral vector pLVshRNA-mCherry(2A)puro (Inovogen Tech, Beijing, China), with scrambled shRNA served as control (shControl). Packaging of pseudo-typed recombinant lentivirus was performed by transfection of 293T cells. Briefly, 1.5 × 10^6^ 293T cells were plated in 6 cm dish and cultured for 20 h. Then the cells were contransfected with either 1.7 μg pLVshRNA-mCherry(2A) puro-shControl or pLVshRNA-mCherry (2A) puro-RSPOs (RSPO1, RSPO2, and RSPO3), 1.13 μg pCMV Δ8.91 and 0.57 μg pMD.G using Lipofectamine 2000 (Invitrogen, Carlsbad, CA, USA). The supernatant containing the lentivirus (Lenti-shRSPO1, Lenti-shRSPO2, Lenti-shRSPO3, Lenti-shDKK1, and Lenti-shControl) was harvested at 72 h, filtered through a 0.45 μm low protein binding filter (Millipore, Bedford, MA), concentrated by centrifugation at 4,000 g for 15 min, and stored at −80°C. Lentiviral vector titers were determined by fluorescence-activated cell sorting analysis, and the viral titer was 1.0 × 10^8^ TU/mL.

**Table 3:** shRNA sequences

Gene		shRNA sequence
**shRSPO1**	Forward	5′-GATCCCCCTGTTCAGAAGTCAACGGTTCAAGAGACCGTTGACTTCTGAAC AGTTTTTTGGAAA-3′
	Reverse	5′-AATTTTTCCAAAAAACTGTTCAGAAGTCAACGGTCTCTTGAACCGTTGACTT CTGAACAGGGG-3′
**shRSPO2**	Forward	5′-GATCCCCGAGAAGGAATGCGTCAGTATCAAGAGTACTGACGCATTCCTTCTC TTTTTTGGAAA-3′
	Reverse	5′-AATTTTTCCAAAAAAGAGAAGGAATGCGTCAGTACTCTTGATACTGACGCATT CCTTCTCGGG-3′
**shRSPO3**	Forward	5′-GATCCCCGGATTGGCATGAAGCAGATTCAAGAGATCTGCTTCATGCCAATCCT TTTTTGGAAA-3′
	Reverse	5′-AATTTTTCCAAAAAAGGATTGGCATGAAGCAGATCTCTTGAATCTGCTTCATG CCAATCCGGG-3′
**shDKK1**	Forward	5′-GATCCCCGAGGTGTACAGATCTGTCTTCAAGAGAGACAGATCTGTACACCTC TTTTTTGGAAA-3′
	Reverse	5′-AATTTTTCCAAAAAAGAGGTGTACAGATCTGTCTCTCTTGAAGACAGATCTGT ACACCTCGGG-3′
**shControl**	Forward	5′-GATCCCCGAGGCATTCTGTTGTACAGTCAAGAGCTCCGTAAGACAACATGTCTT TTTTGGAAA-3′
	Reverse	5′-AATTTTTCCAAAAAAGAGGCATTCTGTTGTACAGCTCTTGACTCCGTAAGACAA CATGTCGGG-3′

HSCs were co-cultured with lentivirual vector (Lenti-shRSPO1, Lenti-shRSPO2, Lenti-shRSPO3, Lenti-shDKK1, and Lenti-shControl severed as the control, MOI = 20). Four days after infection, HSCs were harvested to verify the effect of lentivirus delivery by real-time PCR.

### *In vivo* transduction of lentivirus

*In vivo* transduction of lentiviruses (Lenti-shRSPO1, Lenti-shRSPO2, Lenti-shRSPO3, Lenti-shDKK1, and Lenti-shControl severed as the control) was achieved through tail vein injections of 0.1 mL of concentrated viral suspension with a viral titer of 1.0 × 10^8^ TU/mL lentiviral particles in PBS; injections were conducted once every two weeks. Six weeks after the injection, the animals were sacrificed by CO_2_ exposure and liver tissues were harvested.

### Statistical analysis

Data were presented as mean ± SEM. A two-tailed Student's *t-test* was employed to evaluate the differences between groups. For semi-quantitative analysis of histological staging, nonparametric tests (Wilcoxon test) were used. A value of *p* < 0.05 was considered to be statistically significant.
